# High-Protein Diet Induces Hyperuricemia in a New Animal Model for Studying Human Gout

**DOI:** 10.3390/ijms21062147

**Published:** 2020-03-20

**Authors:** Fan Hong, Aijuan Zheng, Pengfei Xu, Jialin Wang, Tingting Xue, Shu Dai, Shijia Pan, Yuan Guo, Xinlu Xie, Letong Li, Xiaoxiao Qiao, Guohua Liu, Yonggong Zhai

**Affiliations:** 1 Beijing Key Laboratory of Gene Resource and Molecular Development, College of Life Sciences, Beijing Normal University, Beijing 100875, China; hongfanky@126.com (F.H.); PEX9@pitt.edu (P.X.); wangjialin9203@163.com (J.W.); xtt1206@163.com (T.X.); daishu0825@163.com (S.D.); 15129062355@163.com (S.P.); gy5326@126.com (Y.G.); 201921200016@mail.bnu.edu.cn (L.L.); 201921200023@mail.bnu.edu.cn (X.Q.); 2 Key Laboratory for Cell Proliferation and Regulation Biology of State Education Ministry, College of Life Sciences, Beijing Normal University, Beijing 100875, China; 3 Key Laboratory of Feed Biotechnology of Ministry of Agriculture & National Engineering Research Center of Biological Feed, Feed Research Institute, Chinese Academy of Agricultural Sciences, Beijing 100081, China; zhengaijuan77@126.com; 4 Center for Pharmacogenetics and Department of Pharmaceutical Sciences, University of Pittsburgh, Pittsburgh, PA 15213, USA; 5 Beijing First Agriculture Co. LTD, Beijing 100875, China; shsinlou@126.com

**Keywords:** hyperuricemia, high protein diet, gout, chicken, renal disease

## Abstract

Hyperuricemia is a central risk factor for gout and increases the risk for other chronic diseases, including cardiometabolic disease, kidney disease, and hypertension. Overproduction of urate is one of the main reasons for hyperuricemia, and dietary factors including seafoods, meats, and drinking are contributed to the development of it. However, the lack of a suitable animal model for urate metabolism is one of the main reasons for the delay and limitations of hyperuricemia research. Combining evolutionary biological studies and clinical studies, we conclude that chicken is a preferred animal model for hyperuricemia. Thus, we provided chickens a high-protein diet (HPD) to evaluate the changes in the serum urate levels in chickens. In our study, the HPD increased the serum urate level and maintained it at a long-term high level in chickens. Long-term high serum urate levels induced an abnormal chicken claw morphology and the precipitation of monosodium urate (MSU) in joint synovial fluid. In addition, a long-term HPD also decreased the glomerular filtration rate and induced mild renal injury. Most importantly, allopurinol and probenecid displayed the positive effects in decreasing serum urate and then attenuated hyperuricemia in chicken model. These findings provide a novel model for hyperuricemia and a new opportunity to further investigate the effects of long-term hyperuricemia on other metabolic diseases.

## 1. Introduction

Hyperuricemia is a metabolic disease caused by a purine metabolism disorder and is characterized by a serum urate level greater than 420 μmol/L (male) or 360 μmol/L (female) [1]. In the United States, the prevalence of hyperuricemia in adult males and females is 21.2% and 21.6%, respectively [2], and the prevalence was 8.4% in Chinese adults in 2009–2010 [3]. Hyperuricemia is considered an advanced event of gout, which is arthritis caused by excessive urate concentrated at the joint, leading to urate precipitation [4]. Hyperuricemia and gout are important risk factors for many metabolic diseases, including hypertension, cardiovascular diseases, obesity, hyperglycemia, and diabetes. More than 50% of patients with hyperuricemia have obesity, 40%–49.7% of patients with hyperuricemia have hypertension, and 9.5%–13.5% of patients with hyperuricemia also have diabetes [5]. Recently, it was reported that high urate and xanthine levels even became one of the risks of gestational diabetes mellitus [6]. However, the mechanism by which hyperuricemia develops into gout and its effect on other metabolic disorders is not fully understood.

The occurrence of hyperuricemia is the disruption in the homeostasis of purine metabolism. Genetic and environmental factors combined contributed to the development of hyperuricemia and gout. Urate is the end product of human purine metabolism, and mainly produced in the liver, and about 1/3 of the urate is excreted through the intestine and 2/3 of those are excreted by the kidney [7]. However, most of the urate is reabsorbed back into the body during excretion, only about 10% urate is excreted finally in daily [8]. After consuming purine-rich foods by people with normal renal function, urate excreted through the urine was increased by more than 50% compared with those in people without a purine diet, while blood urate levels still remained stable [9]. Hyperuricemia usually occurs due to excessive urate production and decreased excretion [4]. Urate underexcretion has been regarded as the dominant factor in the development of hyperuricemia [10]. Then, ATP binding cassette subfamily G member 2 (ABCG2) is the most widely studied urate transporter, and many studies have identified that this gene generally displayed dysfunctional variants at patients with gout [11,12,13]. However, recent studies have also found that the increased urate excretion load of the kidney is partly due to an increase in purine intake from the diet, and attenuated excretion of urate in the intestines [14]. ABCG2 is also expressed in intestine, and many patients with hyperuricemia lost the expression of ABCG2 in intestines. The current researches based on genome-wide association study (GWAS) analysis almost focus on the comparison between patients and healthy people, and the results displayed the difference in these genes including ABCG2, SLC2A9, HNF1A, HNF4A, ALDH2, CNTN5, MIR302F and so on [15,16]. The mechanism of developing hyperuricemia and gout and the reasons for disruption or mutation in these genes remain unclear. We know that the incidence rate of hyperuricemia and gout, like many metabolic diseases, is increased with aging [17]. However, GWAS analysis is difficult to investigate the effects of environmental factors on urate production and excretion as people aging. In addition, previous studies have suggested that 80% of purine sources are endogenous, but some scholars have provided a novel opinion that dietary purines may disturb the homeostasis of purine metabolism and then be converted to endogenous purine [18]. The abundance rhythmic changes of nucleosides levels in the plasma of mouse also reflect the rapid transformation of dietary purine sources [19]. Therefore, it is important to investigate whether environmental factors induce increased synthesis of urate and whether it will affect intestinal urate excretion.

**Figure 1:**
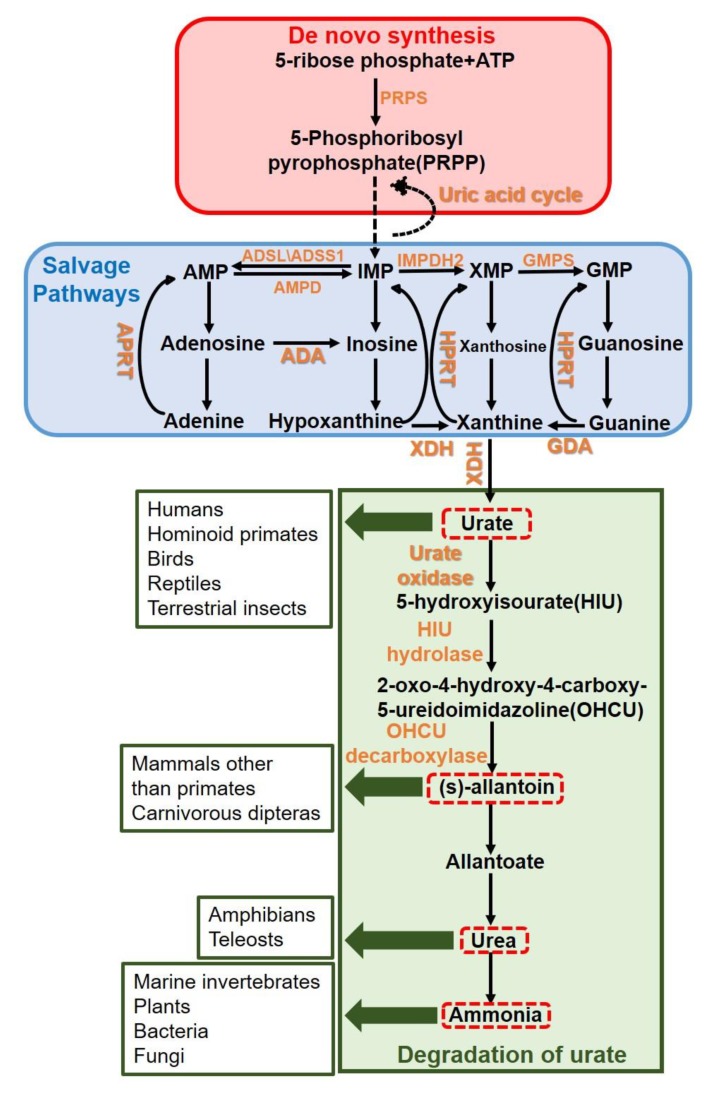
Purine metabolism and urate degradation in different species. Purine metabolism consists of de novo synthesis and salvage pathways. Humans, hominoid primates, birds, reptiles, and terrestrial insects exhibit urate ammonotelism. Mammals other than primates and carnivorous dipteras exhibit allantoin ammonotelism. Amphibians and teleosts exhibit urea ammonotelism. Marine invertebrates, plants, bacteria, and fungi directly excrete ammonia. The arrow from PRPP to IMP means the reactions is complexed and need about 10 steps; the arrow from IMP to PRPP represents the uric acid cycle which is particularly belong to the birds, reptiles and insects; the red dotted boxes represent the four product of urate degradation; the green arrows represent the species which put the correspondence product as end product of urate degradation; the other arrows generally represent one step reaction.

**Figure 2:**
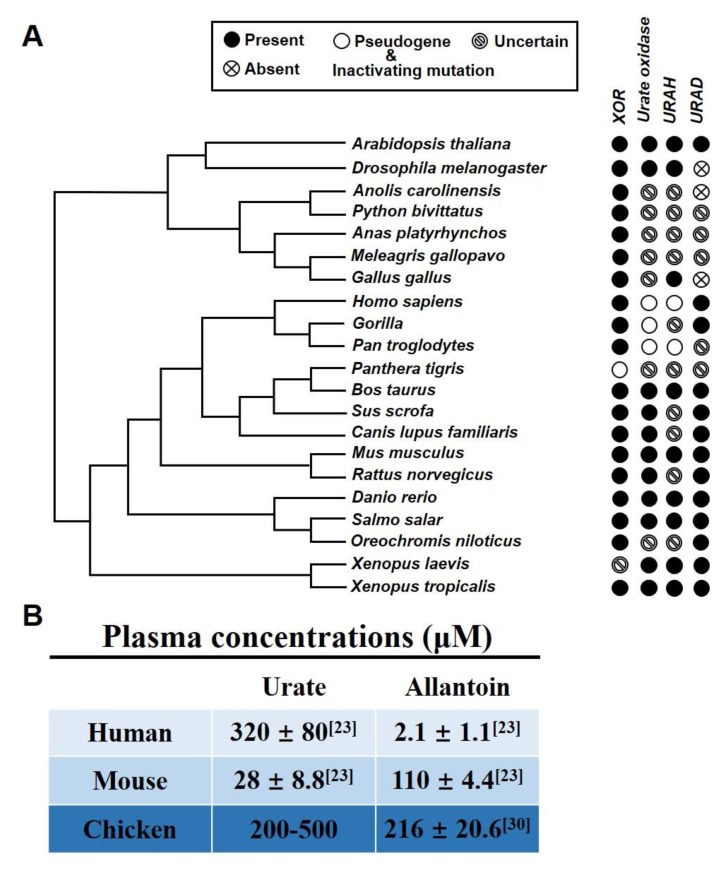
Chicken as a suitable hyperuricemia model among various organisms. (**A**) Phylogenetic tree of urate metabolism-related genes. All participate sequence alignment information was based on database in NCBI gene, the uncertain sign is represented for no related information could obtain in this database. (**B**) Plasma concentrations of urate and allantoin in humans, mice, and chickens. The urate and allantoin level in plasma of human and mouse is referenced from Cantor J et al. [23]; and the level of allantoin is referenced from Carro M.D. et al. [30]; the range value of plasma urate in chicken is from our studies.

The lack of a suitable animal model for urate metabolism is one of the main reasons for the delay and limitation in hyperuricemia research. In an evolutionary biology study, it was found that the uricase in humans and gorillas was mutated 16-24 million years ago; thus, the final product of purine metabolism is urate (Figure 1) [20]. However, other mammals, such as rats, mice, and rabbits, contain uricase and can metabolize urate to allantoin, which is soluble in water and easy to excrete [21]. The key enzymes in urate metabolism, xanthine oxidoreductase (XOR), urate oxidase (UOX), 5-hydroxyisourate (HIU) hydrolase, and 2-oxo-4-hydroxy-4-carboxy-5-ureidoimidazoline (OHCU) decarboxylase are presented in Figure 2A. While there are two forms of XOR: xanthine dehydrogenase (XDH) and xanthine oxidase (XO). The difference between these two forms is dependence on the substrate they used: XDH prefers NAD^+^ and XO prefers O_2_ [22]. In a clinical study, the blood urate level was significantly higher than the blood allantoin level in humans, which contrasts the phenotype in mice (Figure 2B) [23]. In addition, the serum urate level in chickens was also high in our study, although allantoin also exists in chicken blood (Figure 2B) [24]. Thus, chickens could be a potential animal model for hyperuricemia.

Urate is a weak acid that normally exists as monosodium urate (MSU) and has antioxidative abilities, which was needed in the evolution of humans thousands to millions of years ago [20]. In several clinical studies, there was shown a protective effect against neurodegeneration in which higher level urate correlates with decreased risk of developing Parkinson’s disease or amyotrophic lateral sclerosis [25]. As shown by Simoyi M.F. et al., urate is an important antioxidative agent in birds [24]. Furthermore, urate is the end product of ammonia in birds, in which ammonia cannot be synthesized by urea; thus, birds are prone to the accumulation of urate. Moreover, the urate cycle exists in birds, and it is one of the four Krebs cycles in addition to the tricarboxylic acid (TCA) cycle, the urea cycle and the glyoxylate cycle [26], although it is often ignored. In addition, in poultry farming practices, chickens have been shown to have gout [27], which is often associated with an imbalance in diet formulation. Thus, chickens could be a suitable animal model for urate metabolism and gout studies.

Guo X et al. reported the induction of gout in chickens through high-calcium and high-protein diets [28]. Ding X et al. demonstrated that breast cancer resistance protein (*Bcrp*) and multidrug resistance-associated protein 4 (*Mrp4*) mediated urate transport in the kidney and intestine of chicken [29]. Carro M.D. et al. illustrated that allopurinol also played the role in decreasing serum urate in chickens [30]. This drug is commonly used in the clinical treatment of hyperuricemia and gout [31]. By contrast, there are no long-term stable mouse model for hyperuricemia till now [32]. Therefore, in the purine-urate metabolic pathway, chickens are more suitable for studying hyperuricemia and gout disease than rodents, such as mice. However, there is no standard model for hyperuricemia in chickens, and the effect of long-term hyperuricemia on biochemical profiles and different tissues has not yet been investigated. Thus, we decided to establish a suitable chicken model for hyperuricemia with a high-protein diet (HPD) so that further determine the mechanism by which exogenous purine or other factors affect hyperuricemia in future research.

## 2. Results

### 2.1. Chicken as a Suitable Hyperuricemia Model among Various Organisms

To compare the key enzymes associated with urate production and degradation, including XOR, UOX, URAH (HIU hydrolase), and URAD (OHCU decarboxylase), we performed multiple sequence alignment of these four gene sequences by using MEGA X in different species (Figure 2A). Almost all mammals, birds, reptiles, and terrestrial insects have XOR, which indicates that their urate production pathway is dependent on XOR. However, there are many differences in urate degradation enzymes among various species. *Homo sapiens*, *Gorilla gorilla,* and *Pan troglodytes* all had a pseudogene of urate oxidase instead of functional UOX. Interestingly, although all human and hominoid primates lack URAH or have pseudogenes of URAH, *Homo sapiens* and *Gorilla gorilla,* still have URAD. This is a possible reason for the low presence of allantoin in human blood (Figure 2B). More importantly, *Gallus gallus* and other birds and reptiles were all predicted to have the urate oxidase gene but were not predicted to harbor a specific verified functional UOX. In fact, the relatively high serum urate level in chickens was demonstrated by many other studies and our study, and the variation in the urate level of the blood is substantial in different studies. Although there is no evidence that *G. gallus* has urate oxidase, allantoin also exists at a much higher level in *G. gallus* than in humans (Figure 2B). Several studies have demonstrated that birds have another pathway to metabolize urate, mainly due to antioxidation ability of urate [24].

### 2.2. HPD Increases Serum Levels of Urate in Chickens

**Figure 3:**
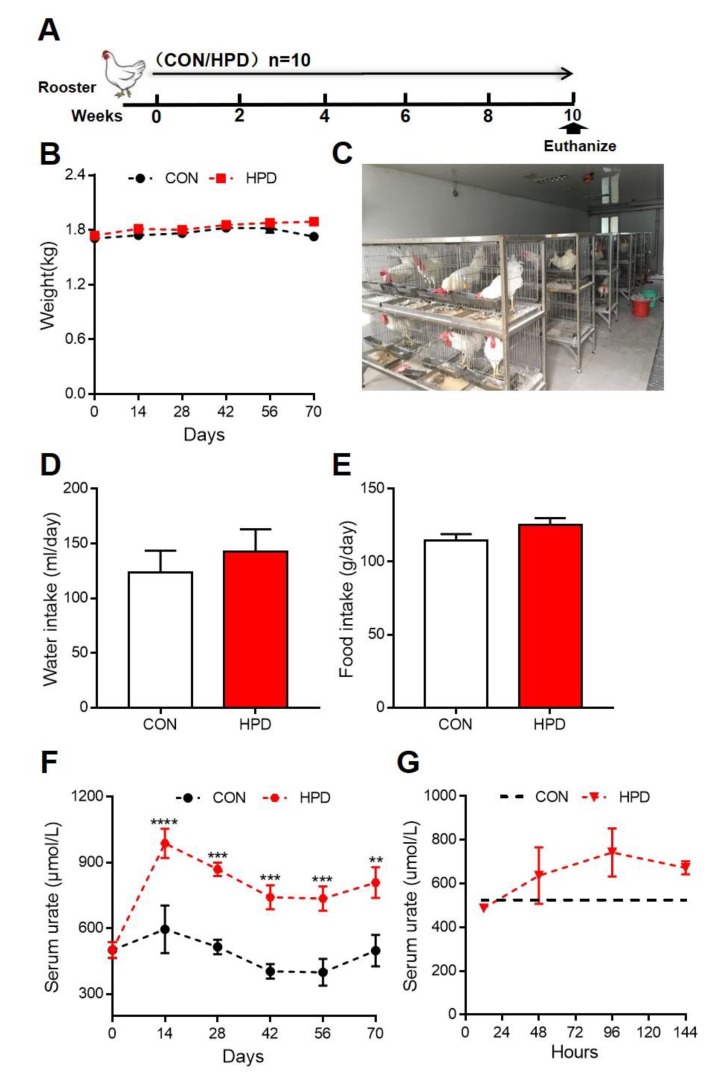
High-protein diet (HPD) increases serum levels of urate in chickens (**A**) Conceptual graph of the treatment group in chickens (*n* = 10). (**B**) Body weight. (**C**) Representative images of the feed condition. (**D**) Water intake. (**E**) Food intake. (**F**) Serum urate levels during consumption for 70 days. (**G**) Serum urate levels at 144 h when chickens were fed HPD (*n* = 6) and dotted line presented the serum urate average value of CON group. Values are presented as the mean ± SEM. Differences were assessed by non-parametric t test followed by Mann–Whitney test and denoted as follows: ** *p* < 0.01, *** *p* < 0.001 and **** *p* < 0.0001 compared with CON.

To establish the long-term hyperuricemia model of chickens, 24-week-old male white leghorns were reared in cages and allowed to consume feed and water ad libitum (Figure 3A,C). Based on different protein contents in the feed: CON (19.11% crude protein) and HPD (34.88% crude protein) were set up (Table 1). During 10 weeks feeding, we detected the weight, serum urate levels, water and food intake changed once every two weeks. There were no significant differences in the weight gain, water intake, and food intake between CON and HPD groups (Figure 3B,D,E). However, the serum urate levels in the HPD group significantly increased compared with those in the CON group after two weeks feeding (Figure 3F). The trend of serum urate in HPD was relatively declined at 28-days and then gradually turned to stable until the end of experiment. It was obvious that HPD consistently increased the serum urate and maintained significantly high-level condition during whole period feeding. To test if the serum urate level changed at the beginning of feeding HPD or not, we detected the serum urate level after changing into HPD feeding at 12, 48, 96, and 144 h in several chickens (*n* = 6). Interestingly, the serum urate levels were increased just after 48-h feeding and gradually increased to approximately 650 μmol/L when chickens have received the HPD 144-h (Figure 3G).

### 2.3. The Effect of HPD on Biochemical Parameters in Chickens

The biochemical parameters of serum in the two groups fed over 70 days are presented in Table 2. Lipid metabolic markers, such as total cholesterol (TC), triglycerides (TG), low-density lipoprotein cholesterol (LDL-C), very-low-density lipoprotein cholesterol (VLDLc), total bilirubin (TB), and direct bilirubin (DB) were decreased in the HPD. This trend was similar to the inhibition of lipid synthesis in human high protein dietary. While, high-density lipoprotein cholesterol (HDL-C), apolipoprotein A-I (ApoA1), apolipoprotein B (ApoB), glucose (GLU), lactate dehydrogenase (LDH), total protein (TP), albumin (ALB), alkaline phosphatase (ALP), P, Ca, K, Na, and Cl were shown to have no difference between the CON and HPD group. These biochemical parameters shown that there was no additional side effect in chickens even in long-term HPD feeding and this dietary induced method was suitable for creation of chicken hyperuricemia model.

### 2.4. HPD Induces an Abnormal Claw Morphology in Chickens

**Figure 4:**
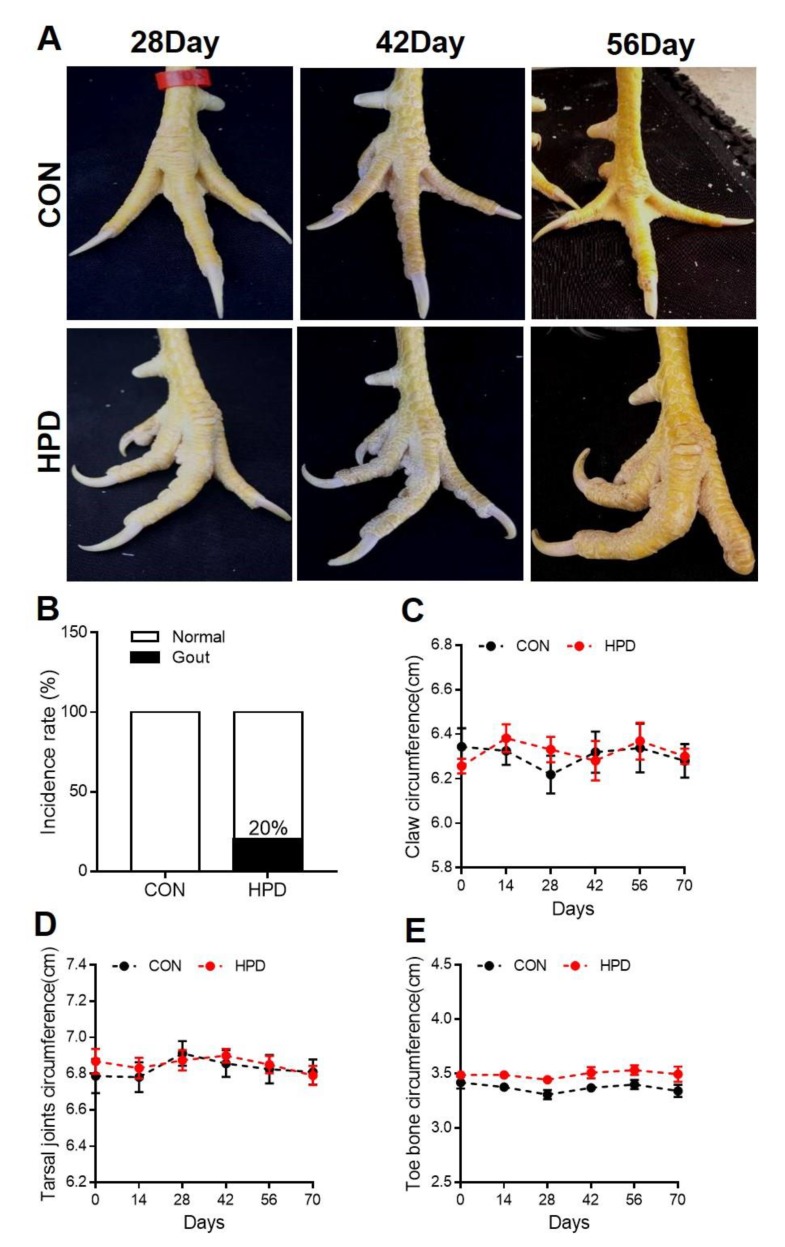
HPD induces an abnormal claw morphology in chickens. (**A**) Representative images of chicken claws after being fed for 28, 42, and 56 days. (**B**) Gout incidence rate. (**C**) Claw circumference, (**D**) tarsal joint circumference, and (**E**) toe bone circumference of chicken. Values are presented as the mean ± SEM.

**Figure 5:**
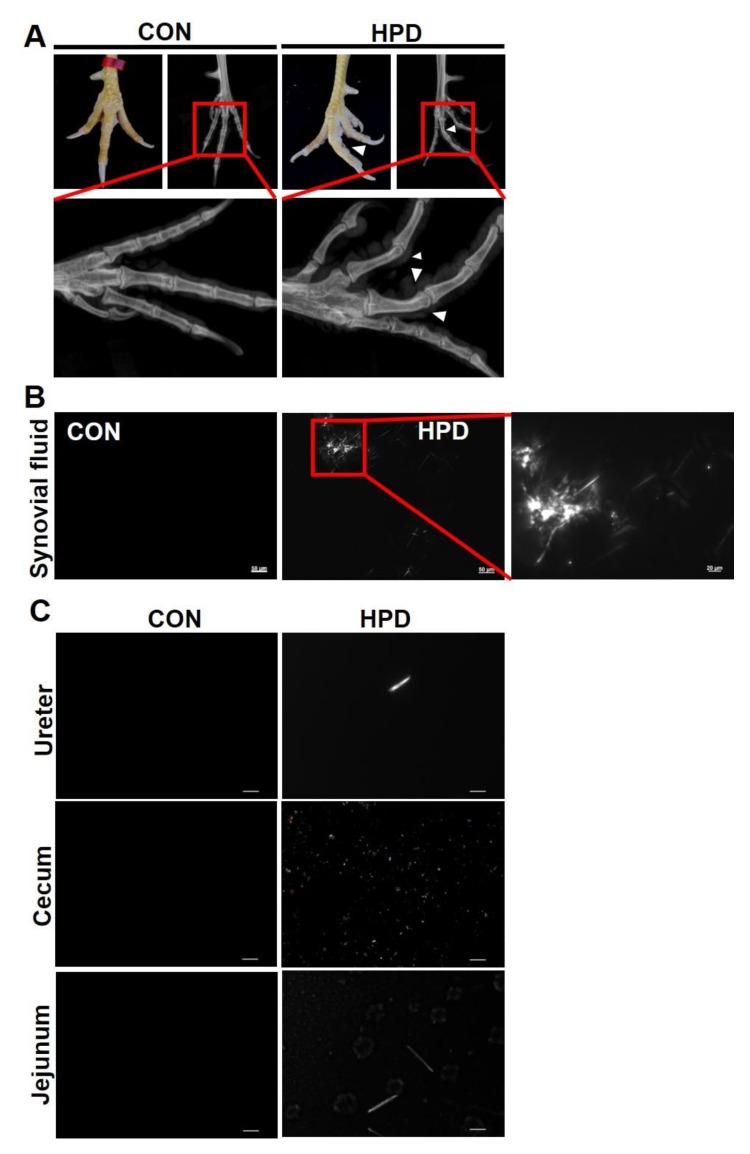
HPD induces monosodium urate (MSU) crystal production in synovial fluid and other tissue fluids. (**A**) Representative images of chicken claws in normal and X-ray conditions. (**B**) Representative images of MSU in synovial fluids using polarized light microscopy. (scale: 50 and 20 µm) (**C**) Representative images of crystal in the ureter, cecum, and jejunum fluids using polarized light microscopy (scale: 20 μm).

After being fed for 10 weeks, some chickens in the HPD group exhibited an abnormal claw morphology (Figure 4A) and the joints of some chickens were bent. Combining the results of MSU detection in synovial fluids of HPD (Figure 5B), the gout incidence rate of HPD group was 20% (Figure 4B). However, this is the case report found in our study and could not entirely represent the gout incidence rate by HPD induced hyperuricemia in chickens. Meanwhile, there were no significant differences in the claw, tarsal joints, and toe bone circumferences between CON and HPD groups (Figure 4C–E).

### 2.5. HPD Induces MSU Crystal Production in Synovial Fluid and Other Tissue Fluids

Next, we further identified the structure of the abnormal claws. We found that the HPD induced the destruction of the toe bone (Figure 5A), which was indicated by X-ray detection. In addition, MSU crystals, as a golden standard in the human gout clinical field, were also detected in the HPD chickens of our study (Figure 5B). There were also MSU crystal detection in ureter tissue fluids and the chyme of the jejunum in the chickens of the HPD group, but we could not exactly identify whether the crystal in the chyme of the cecum is MSU or not (Figure 5C).

### 2.6. HPD Increases the Production of Urate in the Liver

**Figure 6:**
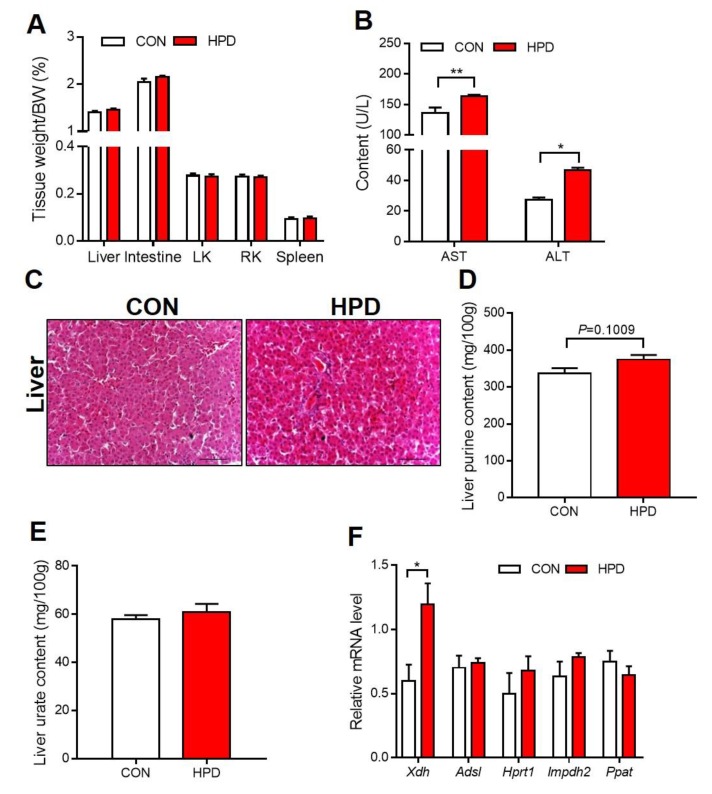
HPD increases the production of urate in the liver. (**A**) Liver, intestine, left kidney (LK), right kidney (RK), and spleen relative weight. (**B**) Serum AST and ALT content. (**C**) H&E staining of liver (scale: 50 μm). (**D**) Liver total purine content, including adenine, guanine, hypoxanthine, and xanthine. (**E**) Liver urate content. (**F**) The expression of urate-producing-related genes including *Xdh*, *Adsl*, *Hprt1*, *Impdh2,* and *Ppat* in the liver. Values are presented as the mean ± SEM. Differences were assessed by non-parametric *t* test followed by Mann–Whitney test and denoted as follows: * *p* < 0.05 and ** *p* < 0.01 compared with CON.

Urate is mainly produced in the liver, and a high-protein or high-purine diet is prone to induce the overproduction of urate in the liver [33,34]. However, there was no significant difference in the liver tissue weights and H&E staining between CON and HPD groups (Figure 6A,C). The liver function markers AST and ALT were significantly increased in the HPD groups (Figure 6B). We further detected the purine and urate content in the liver, and it was mildly increased in the HPD (Figure 6D,E). Urate synthesis-related genes were changed; in particular, *Xdh* significantly increased in the HPD group (Figure 6E).

### 2.7. HPD Induces Renal Injury in Chickens

**Figure 7:**
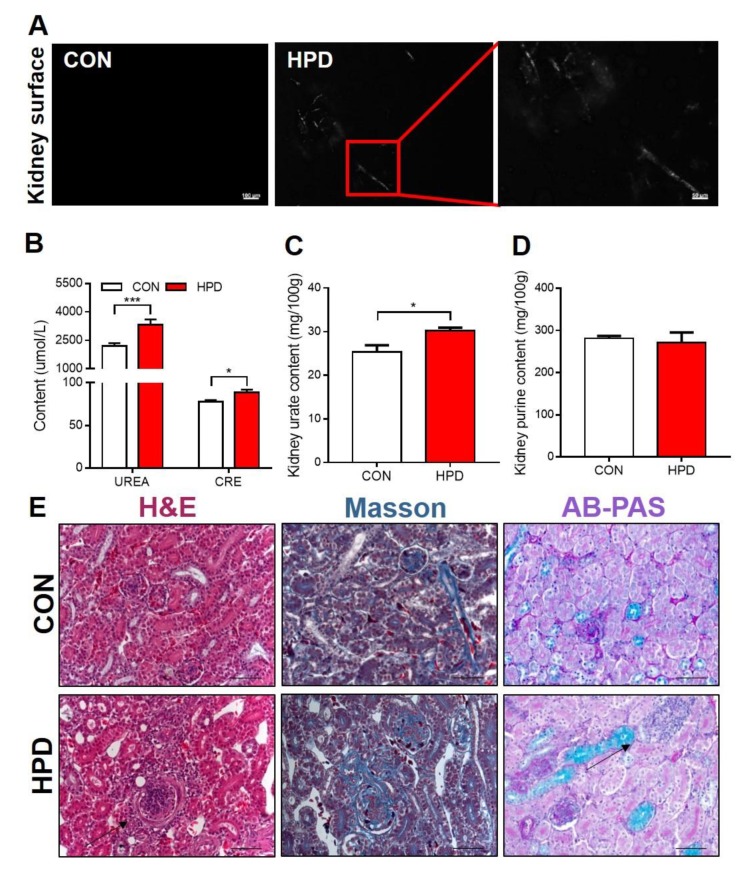
HPD induces renal injury in chickens. (**A**) Representative images of kidney surfaces under polarized light microscopy (scale: 100 μm and 50 μm). (**B**) Serum UREA and CRE content. (**C**) Kidney urate and (**D**) total purine content. (**E**) H&E, Masson, and Alcian Blue Periodic acid Schiff (AB-PAS) staining of kidney (scale: 50 μm). Arrows denote lesions in the kidney. Values are presented as the mean ± SEM. Differences were assessed by non-parametric t test followed by Mann–Whitney test and denoted as follows: * *p* < 0.05 and *** *p* < 0.001 compared with CON.

Hyperuricemia is closely related to renal injury. MSU crystals were detected on the surface of the kidney in the HPD group under polarized brightfield microscopy (Figure 7A). The biochemical parameter serum urea and creatine increased in the HPD group. These parameters are markers of renal injury, possibly due to MSU crystal products in the kidney (Figure 7B). The kidney urate content increased in the HPD group, while the purine content showed no difference between CON and HPD groups (Figure 7C,D). H&E statins showed lesions in the HPD group, in particular, inflammation-like cells enriched around the glomerulus. Masson staining showed no difference between CON and HPD groups. Alcian Blue Periodic acid Schiff (AB-PAS) staining showed mildly PAS-positive reaction, but simultaneously detected the inflammatory cell assembled in the kidney of HFD chickens (Figure 7E).

### 2.8. Allopurinol and Probenecid Treatment Decreases Serum Urate in Chickens of Hyperuricemia

**Figure 8:**
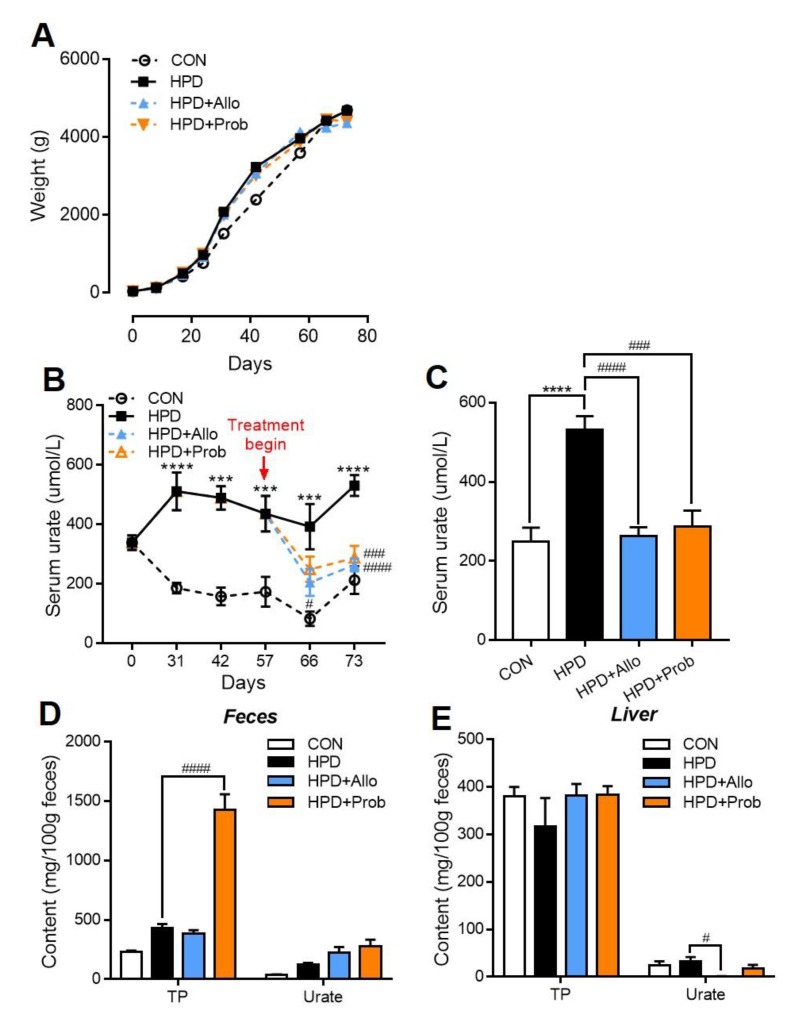
Allopurinol and probenecid treatment decreases serum urate in chickens of hyperuricemia. (**A**) Body weight. (**B**) Serum urate levels during consumption for 73 days, and allopurinol and probenecid treatment began at 57 days and remained until experiment finished. (**C**) Serum urate levels at the end of experiment. (**D**) Feces purine and urate content. (**E**) Liver purine and urate content. Values are presented as the mean ± SEM. *n* = 8–10, *** *p* < 0.001 and **** *p* < 0.0001 compared with CON; # *p* < 0.05 ### *p* < 0.001 and #### *p* < 0.0001 compared with HPD by one-way ANOVA.

Allopurinol and probenecid are widely applied in human hyperuricemia treatment. Thus, we further detect whether these drugs could show a positive effect in the chicken model of hyperuricemia. In AA broiler, HPD still increased the serum urate within 73 days feeding as shown in Figure 8B,C. The relatively decreased trend of serum urate level in CON group during 31 days might be due to more demand of the protein synthesis in chickens which are under a rapid growth phase. Most importantly, allopurinol and probenecid decreased serum urate in chickens from these two drugs treatment began, and this beneficial effect maintained until at the end of experiment (Figure 8B). Probenecid plays the role in decreasing serum urate by increasing urate excretion in human treatment [35], and this effect also found in chickens. As shown in Figure 8D, the purine and urate content were significantly increased in feces of chickens (HPD + Prob group). Another drug allopurinol could inhibit the XOR activity in liver and attenuate the hyperuricemia in clinical [36]. As expected, allopurinol treatment also significantly decreased the serum urate (Figure 8B). In addition, it was shown that urate content was significantly decreased in the liver of HPD + Allo group (Figure 8E). Moreover, the biochemical parameter serum XOR activity showed that the allopurinol treatment inhibited the urate synthesis which was also corresponding to the serum urate data (Table 3).

### 2.9. Allopurinol and Probenecid Treatment Decreases Gout Incidence Rate in Chickens

**Figure 9:**
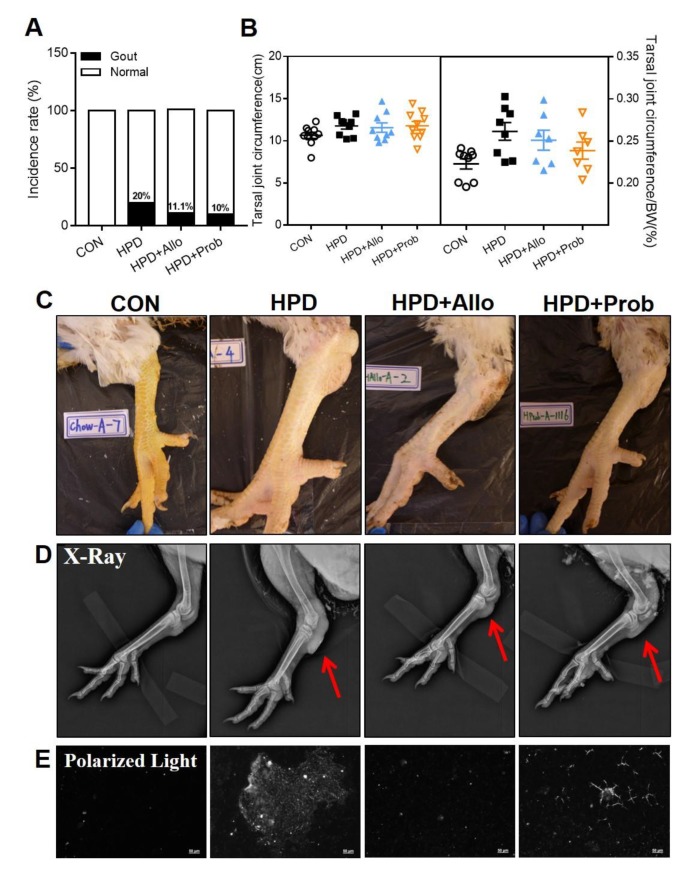
Allopurinol and probenecid treatment decreases gout incidence rate in chickens. (**A**) Gout incidence rate (*n* = 8–10). (**B**) Tarsal joint circumference and tarsal joint circumference/BW of chicken claws. (**C**) Representative images of chicken claws in 73 days. (**D**) Representative images of chicken claws in X-ray conditions. The red arrows are the position of swell in the joint of chickens. (**E**) Representative images of MSU crystal in synovial fluids of chicken claws using polarized light microscopy (scale: 50 µm). Values are presented as the mean ± SEM.

Allopurinol and probenecid treatment also attenuate the gout development in chickens. After 73 days of high-protein feeding, some AA broilers also developed gout, the incidence of gout was 20% in the HPD group (Figure 9A). We found that the AA broilers with gout showed more swelling in the tarsal joint instead of claw joint in white leghorns as presented in Figure 9B–D. More importantly, there was a relatively decreased gout incidence rate in these positive drugs through detecting the circumference of tarsal joint and MSU in synovial fluids in chickens (Figure 9A,B,E). The tarsal joint circumference in these AA broilers was much higher than those in white leghorns and it is due to the weight of chicken (Figure 3B, Figure 4D, Figure 8A and Figure 9B). However, the number of chickens is few, the incidence rate is just a reference. As familiar to white leghorns, lipid metabolic markers including TC and TG in HPD feeding groups (HPD, HPD + Allo, and HPD + Prob) were less than those in CON group. While, serum glucose also decreased in HPD group but not drug treatment groups compared with that in CON group at this time. In addition, serum AST and ALT mildly increased in HPD group but allopurinol and probenecid treatment decreased these biochemical parameters in chickens on the contrast (Table 3).

## 3. Discussion

Hyperuricemia is the outcome of a combination of an endogenous purine metabolism disorder and an exogenous environmental disruption [37]. Recently, a trans-ancestry genome-wide association study, which included more than 450,000 individuals targeted at serum urate demonstrated that 183 loci were associated with gout [15]. However, few studies have focused on exogenous factors, such as dietary factors, that induce hyperuricemia or gout. Commonly, the dietary risks for hyperuricemia or gout are determined by the meta-analysis of population-based cohorts [38]. However, foundational research based on animal models is lacking. One possible reason for this is that mice have uricase to metabolize urate to allantoin and cannot maintain high serum urate levels over a long-term period [39]. A uricase inhibitor called potassium oxonate is widely used in mouse hyperuricemia model. However, it is quickly metabolized by mice within hours and only induces acute hyperuricemia [32]. In addition, the survival rate of UOX knockout mice was approximately 40% in a recent study [40]. While chicken is a preferred animal model for hyperuricemia in similarity of urate metabolism pathway to human. Thus, we would aim to establish a suitable animal model for hyperuricemia with chickens and, further, to deeply investigate the effects of exogenous factors, such as diet, on inducing hyperuricemia.

Evolutionary biology revealed differences in purine metabolism between different species. Only humans and hominoid primates have urate as the end product of purine metabolism [21]. The uricase gene was mutated in the promoter region and protein-coding sequence in multiple independent incidents [20], as it was necessary for humans to increase their antioxidation abilities because the L-gulono-lactone oxidase mutation occurred 40–50 million years ago, which synthesizes vitamin C [41,42,43]. Furthermore, another reason for uricase mutation is that hominids needed urate to increase blood pressure to maintain their vertical position due to low sodium intake in the mid-Pleistocene period (1–2 million years ago) [20,39,44]. Thus, hyperuricemia is closely associated with hypertension [44]. Mammals other than primates, such as mice, rats and rabbits, have uricase that can metabolize urate into HIU, which is unstable and spontaneously hydrolyzed into OHCU and later spontaneously decarboxylated into water-soluble allantoin [45]. Thus, the level of allantoin in blood is much higher than the level of serum urate in these mammals, which contrasts the human serum urate level [23]. In addition, birds, reptiles and terrestrial insects are uricotelic organisms [26]. Interestingly, allantoin also exists in chicken serum, although urate oxidase is not present in birds. This is because there are another series of enzymes that catalyze urate to allantoin [24]. By comparing the important genes involved in urate production, including XOR, UOX, URAH, and URAD, we concluded that chickens are a preferred animal for the hyperuricemia model.

A high-purine diet, which is one of the risk factors for gout, is usually associated with a high-protein diet, such as seafoods, meats, viscera and so on [33,46]. In addition, HPD also elevated oxidative stress in rats and coupled with urate level increased in the cerebral cortex and hypothalamus in rats [47]. In this study, we demonstrated that HPD can increase the serum urate level in white leghorns chickens within 2 weeks, which then gradually declined within 6 weeks and was maintained at a relatively high level until the end of the experiment. To further test the change in the serum urate level at the beginning of HPD feeding, we detected the serum urate levels within 144 h of changing the feed. Serum urate started to increase within 48 h and gradually reached 650 μmol/L at 144 h. Next, we detected biochemical markers in the serum. Interestingly, except for the liver function and kidney function markers that changed in the HPD groups of white leghorns chickens, the cholesterol metabolism-associated markers, such as TC, LDL-C, VLDL-C, TB, and DB, were decreased in the HPD groups. The potential reasons for this phenomenon may be due to the increased protein intake enhancing amino acid metabolism and then producing more acetyl-CoA, which is a source of energy for TCA cycles [26]. These phenotypes were also identified in HPD group of AA broiler. Glucose level also significantly decreased in this breed of chicken when challenged with HPD. Most importantly, long-term high serum urate induced an abnormal claw morphology in several HPD chickens. Further detection of the joints and phalanges of the chickens by X-ray revealed that the joint section had an unclear structure in the HPD group, which was similar to images of joints in mild gout patients [31]. We also detected urate crystals under polarized microscopy in the joint synovial fluid of HPD chickens, which is the gold standard to verify the occurrence of gout in humans [4]. However, the incidence of gout in our study is case report and cannot be entirely accurate due to the limitation in number of chickens. Thus, feeding HPD to chickens not only increased the serum urate levels but also potentially induced gout.

To further test the chicken model whether stable and suitable for drug treatment, we performed other strains of chicken: AA broiler, and feeding with HPD and simultaneously treated with allopurinol and probenecid at 53 days. As expected, HPD also significantly increased serum urate in AA broiler, though the base level of serum urate was different between white leghorns and AA broiler. One of the reasons is that white leghorns we used in the first experiment were much older than AA broiler. Serum urate generally increased with aging in clinical researches [17]. Another reason potentially is the strain difference like biochemical parameters were diverse in different strains of mice. Most important in this experiment was that allopurinol and probenecid shown a well effect in decreasing serum urate in chickens. Allopurinol and probenecid were still used as first-line of drugs in clinical therapies for anti-hyperuricemia [36]. The mechanism for allopurinol is through inhibiting the XOR activity in liver to suppress urate synthesis and then decline serum urate level [4]. The urate content in liver and serum XOR activity were both decreased as followed with serum urate level was significantly decreased in allopurinol treatment in chickens (Figure 8B,C,E). While probenecid plays a role in attenuating hyperuricemia through inhibiting resorption of urate in renal tubule and then increasing the excretion of urate in urine [35]. In chickens, the urine and feces were combined in cloaca, and purine and urate content were significantly increased in feces of chickens which were treated with probenecid (Figure 8D). Additionally, serum creatine and urea level in HPD showed no difference with CON, but serum creatine in HPD + Prob group was relatively higher than that in HPD chickens (Table 3). This phenotype was correspondent to that in human clinical practice, probenecid has some side effects in kidney [4]. These two drugs successfully decreased serum urate in chickens and ever shown a positive effect in inhibiting gout development (Figure 9A,C–E). Thus, the chicken model of hyperuricemia is also displayed suitable characteristic in drug screen research.

It is necessary to investigate the long-term effect of hyperuricemia on other diseases, such as renal diseases, hypertension, diabetes, and obesity. However, few studies have focused on this topic. The production of urate occurs nearly entirely in the liver and is performed by a series of collaborative enzyme reactions [7]. In our study, the HPD induced hyperuricemia in chickens, coupled with the significantly increased serum markers ALT and AST. However, this phenotype was only observed in white leghorns but not in AA broiler, and the basal levels of ALT and AST were obviously different in these two breeds of chickens. Though the significantly increased ALT and AST level in HPD group of white leghorns was seen, no lesions were detected in the liver histology. In fact, birds metabolize amino acids to glutamic acid, glutamine, and aspartate through a urate cycle, in which ALT and AST are key enzymes in this cycle [26]. In addition, the key urate-producing enzyme XDH was significantly increased in the HPD group compared with the CON group and was positively correlated with the serum urate levels. Hyperuricemia is closely associated with kidney excretion disorders [14,48]. We also detected MSU on the kidney surface, which indicates that inflammation occurred in the renal tissue. The marker of glomerular filtration rate is creatine, which was significantly increased in the HPD group of white leghorns. The urea level was also increased in the HPD group of white leghorns. As expected, the urate content increased in the kidney in the HPD group of white leghorns. In histological detection of renal, glomerular interstitial inflammation was detected in the HPD group by H&E and AB-PAS staining, with more inflammatory cell infiltration around the glomerulus. However, there was no difference in Masson’s stain. This phenotype provided insight into hyperuricemia-induced renal-associated diseases.

## 4. Materials and Methods

### 4.1. Phylogenetic Tree Construction

The mutated urate oxidase was searched among different species with the basic local alignment search tool (BLAST) in the National Center for Biotechnology Information (NCBI) database. The FASTA files of these sequences were downloaded, and then multiple sequence alignment was performed by using MEGA X. The neighbor-joining tree was constructed, and the bootstrap test of phylogeny was chosen to modify the phylogenetic trees. In addition, the presence of genes related to urate production and degradation, including XOR, UOX, URAH, and URAD, was evaluated with NCBI searches to identify whether such genes were functional, and these genes were classified as present, pseudogene or inactivating mutation, absent, and uncertain. The uncertain sign is represented for no related information could obtain in NCBI gene database.

### 4.2. Animals and Experimental Design

All chickens were purchased from Beijing Dafa Zhengda Co., Ltd. (Beijing, China). All chickens were reared in cages and allowed to consume feed and water ad libitum and feed in a climate-controlled room at 25~30 °C. The first experiment used male white leghorns at 24-weeks old and then been performed experimental feeding for 10 weeks. They were randomly assigned to two groups (*n* = 10 per group, weight 1800 ± 13.8 g). The normal control diet (CON) was a basal diet containing 19.11% crude protein; HPD consisted of 34.88% crude protein as shown in Table 1. The second experiment used male AA broiler at 1-day old and then been performed experimental feeding for 73 days. They were randomly assigned to four groups (*n* = 10 per group, weight 43.9 ± 0.6 g). The normal control diet (CON) was a basal diet containing 20% crude protein; the HPD consisted of 35% crude protein; HPD + Allo group feeding contains 0.09% allopurinol; HPD + Prob group feeding contains 0.05% probenecid as shown in Table 4. The compositions of the diets were developed based on the National Research Council (1994) recommended requirements and ingredients of feed. All experimental procedures were performed and approved by the guidelines of the Ethics and Animal Welfare Committee of Beijing Normal University (Approval No. CLS-EAW-2020-010; date: 16 March 2020).

### 4.3. Histological Analysis

To make paraffin sections, the liver and kidney were fixed in 4% paraformaldehyde, paraffin-embedded, and sectioned at 4–5 μm. Hematoxylin and eosin (H&E) staining was performed according to standard methods. Masson staining and AB-PAS staining were performed according to the protocols provided with the kits (Beijing Solarbio Science & Technology Co., Ltd., Beijing, China).

### 4.4. Radiographic Imaging

For the analysis, the claws, tibias, and femurs of the chickens were radiographically imaged using MikasaHF100HA (Mikasa X-ray Co., Ltd., Tokyo, Japan) with a Matrx Animal Anesthesia Ventilator System (Midmark Corporation, Torrance, PA, USA) at the end phase of experiment, and the images were processed with Olymmed (Beijing Olymmed Medical Instrument Co., Ltd., Beijing, China).

### 4.5. Biochemical Analysis

Immediately before euthanasia, blood samples were collected from the wing vein. Serum was obtained by centrifugation at 3000× *g* for 10 min. Alanine aminotransferase (ALT), aspartate aminotransferase (AST), creatinine (Cre), urea, total cholesterol (TC), total triglycerides (TG), high-density lipoprotein cholesterol (HDL-C), low-density lipoprotein cholesterol (LDL-C), very-low-density lipoprotein cholesterol (VLDLc), apolipoprotein A I (ApoA1), apolipoprotein B (ApoB), glucose (GLU), lactate dehydrogenase (LDH), γ-glutamyltransferase (γ-GT), total protein (TP), albumin (ALB), alkaline phosphatase (ALP), total bilirubin (TB), direct bilirubin (DB), xanthine oxidase (XOR), phosphorus, calcium, potassium, sodium, and chloride levels were measured using a Hitachi 7600 automated biochemical analyzer (Diamond Diagnostics Inc., Holliston, MA, USA). Serum urate levels were determined with a urate detection kit (Nanjing Jiancheng Bioengineering Institute, Nanjing, China).

### 4.6. Gene Expression Analysis

Total RNA of tissues was extracted by using an RNAprep Pure tissue kit (Tiangen, Beijing, China). The step of reversing transcribed to cDNA from total RNA was using M-MLV transcriptase (Promega, Madison, WI, USA). The SYBR Green qPCR SuperMix (Transgen Biotech, Beijing, China) was used to perform quantitative real-time PCR on an ABI Q6 instrument (Thermo Fisher Scientific, Waltham, MA, USA). All genes expression in this study was to takeglyceraldehyde-3-phosphate dehydrogenase (*Gapdh*) as reference genes. The primers we used are listed in Table 5.

### 4.7. High-Performance Liquid Chromatography Analysis (HPLC) Analysis

To detect the urate and purine contents of the liver, kidney and feces, 200 mg of sample was added to 5 mL trifluoroacetic acid, 5 mL formic acid, and 10 mL H_2_O and hydrolyzed at 90 °C for 15 min. After hydrolysis, the hydrolysate was cooled quickly, and the solvent was dried using a vacuum centrifugal concentrator (Thermo Fisher Scientific, Waltham, MA, USA). The sample was redissolved in the mobile phase and filtered through a 0.45 μm filter disk. HPLC analysis was performed using an Agilent 1200 series HPLC System. Sample solution (20 μL) was injected onto an Aq-C18 column (4.6 mm × 250 mm, 5 μm) (Agilent, Santa Clara, CA, USA). Elution was carried out using 0.007 mol/L KH_2_PO_4_-H_3_PO_4_. The column was maintained at room temperature. The flow rate was 1 mL/min, and detection was performed at 254 nm.

### 4.8. MSU Crystal Detection

After euthanasia, the claw synovial and ureter fluids and the cecum and jejunum chyme were evaluated via polarized brightfield microscopy with a ZEISS ImagerM1 microscope (Carl Zeiss AG, Oberkochen, Germany).

### 4.9. Statistical Analysis

The results are presented as the mean ± SEM. The statistical analysis was performed using SPSS, version 20 (IBM, Armonk, NY, USA). Differences between groups were statistically analyzed using non-parametric t test followed by Mann–Whitney test or one-way ANOVA by Kruskal–Wallis test were considered statistically with a level of *p* < 0.05.

## 5. Conclusions

In conclusion, evolution biology and current clinical serum urate levels confirm the close relationship between humans and chickens in urate metabolism. In our study, we demonstrated that HPD could induce an increase in the serum urate level in chickens and long-term hyperuricemia. In addition, long-term hyperuricemia also has the potential to induce gout and renal disorders in our chicken models. Allopurinol and probenecid treatment also displayed positive effects in hyperuricemia model of chicken. Thus, establishing a chicken model for hyperuricemia is suitable and feasible for further hyperuricemia and gout research.

## Figures and Tables

**Table 1:** Feed ingredients of first experiment.

Ingredient	CON	HPD
Corn (8.7%)	55.07	38.55
Soyabean Meal (GB2)	26.81	48.07
Rice Bran (GB2)	3.00	2.10
Wheat Bran (GB1)	3.00	2.10
NaCl	0.35	0.25
CaHPO_4_	1.57	1.10
Limestone	9.37	7.26
dl-Methionine	0.10	0.07
Choline	0.10	0.07
Compound Vitamin Premix	0.03	0.02
Trace Element Premix	0.30	0.21
Betaine	0.30	0.21
Crude Protein (%)	19.11	34.88
Lysine (%)	0.94	0.66
Methionine (%)	0.35	0.25
Methionine + Cystine (%)	0.66	0.46
Calcium (%)	3.81	2.95
Availed Phosphorus (%)	0.36	0.25
Metabolic Energy (Kcal/kg)	2650	2650

Corn contains 8.7% crude protein; GB2, quality of soybean meal and rice bran within the Chinese national standard 2; GB1, quality of wheat bran within the Chinese national standard 1. Calculated metabolic energy, crude protein, lysine, methionine, cystine, calcium, and available phosphorus are listed in the table.

**Table 2:** Biochemical parameters in the first experiment chicken model of hyperuricemia.

Parameter	CON	HPD
TC (mmol/L)	3.14 ± 0.43	2.45 ± 0.41 ^a^
TG (mmol/L)	1 ± 0.23	0.69 ± 0.19 ^a^
HDL-C (mmol/L)	1.77 ± 0.3	1.56 ± 0.29
LDL-C (mmol/L)	0.72 ± 0.08	0.49 ± 0.09 ^a^
VLDLc (mmol/L)	0.65 ± 0.16	0.4 ± 0.08 ^a^
ApoA1 (g/dL)	0.11 ± 0.02	0.12 ± 0.02
ApoB (g/dL)	0.22 ± 0.04	0.23 ± 0.03
GLU (mmol/L)	8.72 ± 1.14	8.8 ± 1.23
LDH (U/L)	745.42 ± 46.02	609.27 ± 98.52
TP (g/L)	51.55 ± 4.73	51.2 ± 2.64
ALB (g/L)	17 ± 0.78	17.38 ± 0.64
ALP (U/L)	691.62 ± 72.07	722.83 ± 95.45
TB (μmol/L)	12.54 ± 2.72	5.8 ± 1.47 ^a^
DB (μmol/L)	2.83 ± 0.67	1.7 ± 0.39 ^a^
P (mmol/L)	1.3 ± 0.18	1.27 ± 0.1
Ca (mmol/L)	2.9 ± 0.09	2.95 ± 0.02
K (mmol/L)	5.18 ± 0.48	5.1 ± 0.76
Na (mmol/L)	145.17 ± 5.34	141.67 ± 3.88
Cl (mmol/L)	106.33 ± 4.08	109.67 ± 4.76

The data are expressed as the mean ± SEM of *n* = 6 chickens per group compared with the CON group (^a^, *p* < 0.05) using non-parametric t test followed by Mann–Whitney test. TC, total cholesterol; TG, triglycerides; HDL-C, high-density lipoprotein cholesterol; LDL-C, low-density lipoprotein cholesterol; VLDLc, very-low-density lipoprotein cholesterol; ApoA1, apolipoprotein A-I; ApoB, apolipoprotein B; GLU, glucose; LDH, lactate dehydrogenase; TP, total protein; ALB, albumin; ALP, alkaline phosphatase; TB, total bilirubin; DB, direct bilirubin; P, phosphorus; Ca, calcium; K, potassium; Na, sodium; Cl, chlorine.

**Table 3:** Biochemical parameters in the second experiment chicken model of hyperuricemia.

Parameter	CON	HPD	HPD + Allo	HPD + Prob
TC (mmol/L)	3.58 ± 0.41	2.45 ± 0.31 ^a^	3.04 ± 0.67	3.04 ± 0.94
TG (mmol/L)	1.11 ± 0.47	0.58 ± 0.12 ^a^	0.61 ± 0.15 ^a^	0.62 ± 0.25 ^a^
ALT(U/L)	6.01 ± 1.11	5.51 ± 1.24	4.56 ± 0.92 ^a^	4.42 ± 0.68 ^a^
AST(U/L)	520 ± 169.54	649.72 ± 301.58	466.07 ± 127.14	356.27 ± 92.24 ^b^
XOR(U/L)	5.15 ± 1.46	7.26 ± 1.99 ^a^	5.82 ± 1.02 ^b^	6.79 ± 2.51 ^b^
GLU (mmol/L)	5.3 ± 1.31	2.43 ± 1.13 ^a^	4.2 ± 2.34	6.49 ± 1.66 ^b^
γ-GT (U/L)	6.24 ± 1.93	6.27 ± 1.55	7.24 ± 2.57	7.17 ± 1.57
UREA (mmol/L)	0.27 ± 0.09	0.26 ± 0.07	0.28 ± 0.06	0.26 ± 0.04
CREA (μmol/L)	17.53 ± 3.16	19.18 ± 3.65	18.48 ± 2.7	22.19 ± 4.28

γ-GT, γ-glutamyl transpeptidase. The data are expressed as the mean ± SEM of *n* = 6 chickens per group compared with the CON group (^a^, *p* < 0.05); chickens per group compared with the HPD group (^b^, *p* < 0.05) using one-way ANOVA, followed by Kruskal–Wallis test.

**Table 4:** Feed ingredients of second experiment.

Ingredient	CON	HPD	HPD + Allo	HPD + Prob
Corn (GB2)	65.00	27.00	27.00	27.00
Fish Meal	9.71	10.00	10.00	10.00
Beer Yeast	19.61	65.52	65.52	65.52
Soya Oil	2.39	5.00	5.00	5.00
L-Lysine	0.04	0.00	0.00	0.00
DL-Methionine	0.10	0.00	0.00	0.00
Stone Powder	1.39	1.10	1.10	1.10
CaHPO_4_·2H_2_O	0.06	0.00	0.00	0.00
Compound Premix	0.30	0.30	0.30	0.30
Zeolite Powder	1.29	0.00	0.00	0.00
Crude Protein (%)	20.00	35.00	35.00	35.00
L-Lysine (%)	1.25	2.45	2.45	2.45
DL-Methionine (%)	0.55	0.67	0.67	0.67
Calcium (%)	0.95	0.95	0.95	0.95
Available Phosphorus (%)	0.45	0.61	0.61	0.61
Salinity (%)	0.35	0.40	0.40	0.40
Methionine + cysteine (%)	0.80	1.10	1.10	1.10
Allopurinol (%)	0.00	0.00	0.09	0.00
Probenecid (%)	0.00	0.00	0.00	0.05
Metabolic Energy (Kcal/kg)	3000	2910	2910	2910

Corn contains 8.7% crude protein; GB2, quality of soybean meal and rice bran within the Chinese national standard 2. Calculated metabolic energy, crude protein, lysine, methionine, calcium, and available phosphorus are listed in the table.

**Table 5:** Sequences of the primers used for RT-PCR.

Gene	Forward (5′-3′)	Reverse (5′-3′)	Product Length (bp)
*Xdh*	GAGGGATTTACTCTACGGCGA	AGCCTGATTCAGAAACGGGAC	174
*Adsl*	CAAAGCTGCAGCCATCATTCACCT	GAGCTTGGGCAGCAGCAAGTTAAA	106
*Hprt1*	TGGACTTGTTCTGCATACCCAA	TCATCCCCACCAATGACTTTTA	307
*Impdh2*	GCTGGTTGGCATCATCTCAT	GCCGGTACTTGTCATCTTCG	320
*Ppat*	CGACACTGCTGACTGGGTA	TACTGGAATAAGGCGACCA	151
*Gapdh*	CATGGTTGACACCCATCACAA	GGTGCTAAGCGTGTTATCATCTCA	70
